# Potential impact and cost-effectiveness of rotavirus vaccination in Afghanistan

**DOI:** 10.1016/j.vaccine.2017.10.058

**Published:** 2018-12-14

**Authors:** Palwasha Anwari, Frederic Debellut, Clint Pecenka, Sardar M. Parwiz, Andrew Clark, Devin Groman, Najibullah Safi

**Affiliations:** a Afghanistan National Immunization Technical Advisory Group, District 10, Kabul, Afghanistan; b PATH, Rue de Varembé 7, 1202 Geneva, Switzerland; c PATH, 2201 Westlake Ave, Suite 200, Seattle, WA 98121, USA; d Expanded Program on Immunization, Directorate General of Preventive Medicine, Ministry of Public Health, District 1, Afghanistan; e London School of Hygiene and Tropical Medicine, Keppel Street, London WC1E 7HT, United Kingdom; f Directorate General of Preventive Medicine, Ministry of Public Health, Masood Square, District 10, Kabul, Afghanistan

**Keywords:** Rotavirus, Rotavirus vaccine, Cost-effectiveness, DALY, Incremental cost-effectiveness ratio, Afghanistan

## Abstract

**Introduction:**

Despite progress made in child survival in the past 20 years, 5.9 million children under five years died in 2015, with 9% of these deaths due to diarrhea. Rotavirus is responsible for more than a third of diarrhea deaths. In 2013, rotavirus was estimated to cause 215,000 deaths among children under five years, including 89,000 in Asia. As of April 2017, 92 countries worldwide have introduced rotavirus vaccination in their national immunization program. Afghanistan has applied for Gavi support to introduce rotavirus vaccination nationally. This study estimates the potential impact and cost-effectiveness of a national rotavirus immunization program in Afghanistan.

**Methods:**

This study examined the use of Rotarix® (RV1) administered using a two-dose schedule at 6 and 10 weeks of age. We used the ProVac Initiative’s UNIVAC model (version 1.2.09) to evaluate the impact and cost-effectiveness of a rotavirus vaccine program compared with no vaccine over ten birth cohorts from 2017 to 2026 with a 3% annual discount rate. All monetary units are adjusted to 2017 US$.

**Results:**

Rotavirus vaccination in Afghanistan has the potential to avert more than one million cases; 660,000 outpatient visits; approximately 50,000 hospital admissions; 650,000 DALYs; and 12,000 deaths, over 10 years. Not accounting for any Gavi subsidy, rotavirus vaccination can avert DALYs at US$82/DALY from the government perspective and US$80/DALY from the societal perspective. With Gavi support, DALYs can be averted at US$29/DALY and US$31/DALY from the societal and government perspective, respectively. The average yearly cost of a rotavirus vaccination program would represent 2.8% of the total immunization budget expected in 2017 and 0.1% of total health expenditure.

**Conclusion:**

The introduction of rotavirus vaccination would be highly cost-effective in Afghanistan, and even more so with a Gavi subsidy.

## 1 Introduction

Despite a 53% reduction in the rate of all-cause mortality worldwide since 1990, 5.9 million children under five years died in 2015, with 9% of these deaths due to diarrhea [1]. In the most recent Global Burden of Disease (GBD) 2015 study, diarrheal diseases were the fourth leading cause of death among children under five years, responsible for 499,000 deaths, representing 8.6% of all deaths in this age group [2]. Rotavirus is responsible for more than a third of these deaths [2]. In 2013, the World Health Organization (WHO) and Centers for Disease Control and Prevention (CDC) estimated 215,000 deaths among children under five years, including 89,000 in Asia [3]. Rotavirus infects nearly every child by age 5 and is the leading cause of severe, dehydrating diarrhea in most low-income countries in Asia and Africa [4].

The GBD 2015 study estimated 103,692 child deaths due to diarrhea in the Eastern Mediterranean Region (EMR) and 13,180 deaths due to rotavirus [5], [6]. The Afghanistan Demographic and Health Survey (DHS) 2015 reported that 29% of children under five years had diarrhea in the two weeks before the survey [7]. Surveillance data reported that more than 45% of all hospitalizations for gastroenteritis in the same population in the country are attributable to rotavirus [8]. In addition, Afghanistan is one of the ten countries that account for almost two thirds of all rotavirus deaths in 2013 [3]. All of this evidence points to significant rotavirus burden in Afghanistan and the region.

There are currently two WHO prequalified live oral attenuated vaccines available to countries to help abate the burden of rotavirus disease. These vaccines are the monovalent (RV1) Rotarix® (GlaxoSmithKline Biologicals, Rixensart, Belgium) and the pentavalent (RV5) RotaTeq® (Merck & Co. Inc., West Point, PA, USA) [9]. Gavi, the Vaccine Alliance, provides donor support to help eligible countries introduce these vaccines into their national immunization programs [10]. As of April 2017, 92 countries worldwide have introduced rotavirus vaccine in their national immunization program. Afghanistan introduced Hib vaccines in 2009, pneumococcal conjugated vaccines (PCV) in 2013 and inactivated polio vaccine (IPV) in 2015 [11]. Discussions around introducing rotavirus vaccines have been ongoing for several years and the country decided to apply for support from Gavi in 2017. Gavi has approved Afghanistan’s application and rotavirus vaccine introduction is planned for the first quarter of 2018.

This study evaluates the projected impact and cost-effectiveness of a national rotavirus vaccine introduction in Afghanistan and provides estimates of its cost. The aim is to help policy makers and program managers make informed decisions about the allocation of health resources in Afghanistan.

## 2 Materials and methods

This study examined the use of Rotarix® (RV1), which would be administered following a two-dose schedule at 6 and 10 weeks of age alongside Penta1 and Penta2, respectively. We evaluated the impact and cost-effectiveness of a rotavirus vaccine program compared with no vaccine, at the national level, over ten birth cohorts starting in 2017. Rotavirus disease events and treatment costs are estimated over the first 5 years of life. The estimated average life expectancy of each birth cohort is used to estimate healthy future years of life gained. Future health benefits and costs are discounted using a 3% annual rate. We examine results from the government and societal perspectives. All monetary units were adjusted to 2017 US$.

The primary outcome measure of the analysis is the discounted cost per disability adjusted life year (DALY) averted. Other outcomes include the incremental cost of the vaccine program, as well as the number of averted cases, visits, hospitalizations, treatment costs, and deaths.

### 2.1 Model

We used UNIVAC (version 1.2.09), a model developed at the London School of Hygiene and Tropical Medicine which builds on previous tools collaboratively developed by the Pan American Health Organization’s (PAHO) ProVac Initiative, GAVI’s Hib Initiative, PATH, and the CDC [12]. UNIVAC is a single universal vaccine impact and cost-effectiveness decision support model developed in a standardized, accessible Excel-based interface (Excel, Microsoft Corp, Redmond, WA, US). This model is being used for the first time in Afghanistan to evaluate the impact and cost-effectiveness of rotavirus vaccination. UNIVAC’s precursor, TRIVAC, has been used in many studies worldwide and shares many similarities with UNIVAC [13]. In brief, UNIVAC is a deterministic static cohort model that can be used to generate transparent and conservative estimates of the projected impact of new vaccines. It has been purposefully designed for use in low- and middle-income countries, by national teams working in conjunction with the Ministry of Health. Model input parameters include demographics, burden of disease, vaccine schedule, vaccine efficacy, vaccine coverage, vaccine timeliness, vaccine program costs, and health service costs. Input parameters were gathered from the literature and local sources and then discussed by a group of local experts convened specifically to participate in the cost-effectiveness analysis by providing insight and validating the main assumptions used. This group was comprised of experts from the directorate general of preventive medicine, the health economics and financing department (HEFD), the national expanded program on immunization (EPI), and the health management and information system (HMIS) unit of the Afghan Ministry of Public Health (MoPH).

### 2.2 Disease burden

#### 2.2.1 Incidence and severity

We estimated the incidence of rotavirus disease cases in children under five years to be approximately 10,000 per 100,000 per year, based on a global systematic review and meta-analysis study by Bilcke et al. [14]. This incidence level is consistent with the incidence observed by Zaman et al., in the placebo arm of the RotaTeq randomized controlled trial in Bangladesh and Vietnam [15]. We assumed that 17.25% of the rotavirus disease cases would be severe based on the mean proportion reported for Bangladesh, India, Nepal, and Pakistan in the Platts-Mills MAL-ED study [16]. We therefore assumed 1725 severe rotavirus disease cases per 100,000 per year, and 8275 non-severe rotavirus disease cases per 100,000 per year, in children under five years. The Afghanistan Demographic and Health Survey (DHS) 2015 reported that 54.2% of children with diarrhea were taken to a medical health facility or healthcare provider for advice or treatment [7]. We therefore assumed that 935 severe cases and 4485 non-severe cases would visit a healthcare facility for outpatient treatment, per 100,000 per year in children under five years. In addition, the number of severe rotavirus disease cases resulting in an inpatient visit was estimated from routine HMIS data to be 403 per 100,000 per year in children under five years, implying an overall treatment seeking rate of nearly eighty percent for severe cases [17]. Estimated weighted DALYs for moderate and severe diarrhea in 2013 were 0.188 and 0.247, respectively [18]. When calculating DALYs, we assumed the average duration of rotavirus disease to be 7 days for severe cases and 3 days for non-severe cases [19].

#### 2.2.2 Mortality

There are no robust data on mortality attributed to rotavirus in Afghanistan. We used a rotavirus disease mortality rate of 98 deaths per 100,000 per year in children under five years, as estimated by Tate et al. [3]. To account for uncertainty, we also looked at a high mortality scenario (applying a 15% increase to this mortality rate) as well as a scenario accounting for a rotavirus mortality rate as low as 27.83 deaths per 100,000 per year in children under five years, as reported by Institute for Health Metrics and Evaluation (IHME) in GBD 2015 [20].

### 2.3 Vaccine coverage and efficacy

Because Afghanistan anticipates delivery of rotavirus vaccine alongside Pentavalent vaccine, we used coverage data from Penta (77.3%) reported from the national immunization coverage survey 2013 [21] to project expected coverage rates of the rotavirus vaccination program. Because DHS data from 2015 reports a lower coverage rate of Penta, in an alternative scenario, we also looked at a different coverage projection, using a lower initial rate [7]. Coverage projections are in line with the MoPH strategy to reach 90% immunization coverage of dose one for all antigens nationally by 2020 [22], [23]. We assumed that timeliness of the first two doses of rotavirus vaccine would be similar to the timeliness observed for Penta1 and Penta2 [21]. Based on recommendations of the WHO SAGE, we did not apply the age restriction to vaccination schedule in this analysis [9].

Vaccine efficacy for severe and non-severe cases in the first year following vaccination was estimated based on findings of a randomized, double blind, placebo-controlled trial study of Rotateq vaccination conducted in Bangladesh in 2010. We assumed vaccine efficacy for the two-dose schedule to be 53.1% and a single dose was conservatively assumed to provide half that efficacy [15], [24]. We assumed rates of waning vaccine-induced protection consistent with the waning observed in the trial. We conservatively excluded any indirect benefits of vaccination such as herd effects. Input parameters and sources of data used for estimating disease burden are shown in Table 1.

**Table 1:** Input parameters and source of data for estimation of disease burden.

Parameter	Estimate	Reference(s)
*Age-specific rates per 100,000 per year among children aged 1–59 months*		
Non-severe RVGE cases	8275	#14 and #16
Non-severe RVGE visits	4485	Derived from #7
Severe RVGE cases	1725	#14 and #16
Severe RVGE visits	935	Derived from#7
Severe RVGE hospitalized	403	#5 and #7
Severe RVGE deaths	98	#3

*Disability weight for DALY calculations*		
Rotavirus (non-severe) case	0.188	#18
Rotavirus (severe) cases	0.247	#18

*Mean duration of illness (in days)*		
Rotavirus (non-severe) case	3	#19
Rotavirus (severe) cases	7	#19

*Age distribution of illness cases*		
Age distribution	Cumulative percentage	#8
<1 month	0.4%	
<2 months	5.1%	
<3 months	11.4%	
<6 months	27.1%	
<1 year	69.4%	
<2 years	92.8%	
<3 years	98.0%	
<4 years	99.2%	
<5 years	100%	

### 2.4 Vaccine price and delivery cost

In the base case scenario, we used the last known vaccine price secured by Gavi of US$2.02 per dose [25]. We assumed vaccine price would remain constant at US$2.02 over 10 years (2017–2026) [26]. Based on the Gavi eligibility and transition policy, Afghanistan falls in the initial self-financing transition phase where the minimum co-financing required is US$0.20 per dose with no annual increase as we do not anticipate Afghanistan’s co-financing share to change during the period of analysis [23], [27]. We applied this co-financing amount per dose in alternative scenarios.

We used a wastage rate of 8% as reported for PCV [28], a proxy for rotavirus vaccine wastage, and applied 5% and 7% of the vaccine price to account for international handling and delivery, respectively [23]. We accounted for US$0.80 as a unit cost for safety bags which have a capacity of 100 doses [23].

To account for additional (incremental) costs of delivering the vaccine, we used cMYP data for the year 2017 and estimated the share (US$1.06) that would be incremental for the delivery of the rotavirus vaccine. This amount reflects the average cost per dose of vaccines delivered by the routine immunization program accounting for cost of personnel, transportation, cold chain, other capital equipment and other routine recurrent costs. While we use the average cost per dose delivered for this calculation, we believe the average cost for these categories is representative of the incremental cost of adding a new vaccine. We also explored a higher, doubled incremental delivery cost in an alternative scenario.

### 2.5 Health service costs

The costing of the Basic Package of Health Services (BPHS) assessment in Afghanistan in 2016 estimated a cost of US$1.70 per health facility visit for a diarrhea episode [29]. Similarly, the facility cost of an inpatient case was estimated to be US$8.80. To estimate household costs, we used secondary data from a publication by Rheingans et al. [30], using the household costs associated with childhood diarrhea for Bangladesh as a proxy for those in Afghanistan (Table 2).

**Table 2:** Input parameters for estimating health services costs (all costs are presented in 2017 US$).

Parameter	Government cost	Households costs	Society cost	Reference(s)
Non-severe cases (facility outpatient)	1.72	1.97	3.69	#30 and #31
Severe cases (facility outpatient)	1.72	7.08	8.80	#30 and #31
Severe cases (hospitalization)	8.80	7.14	15.94	#30 and #31

### 2.6 Scenario analysis

In UNIVAC, base case results are calculated using our best estimates of each input parameter. However, each parameter introduces an additional range of uncertainty. A scenario analysis is a way to deal with uncertainties around model parameter estimates and assumptions. This form of uncertainty analysis helps to evaluate different policy scenarios and/or ranges of parameter estimates by creating a series of *‘what if’* scenarios. The range of results provided by comparing the base case to alternative scenarios can be a powerful input to policy discussions, particularly where scenarios with inputs that are unfavorable to the vaccine (e.g. low mortality, no herd effects, no Gavi subsidy) are still shown to generate cost-effective results.

## 3 Results

### 3.1 Base case scenario

Cost-effectiveness results are presented using the discounted incremental cost-effectiveness ratio (ICER). This outcome is calculated by dividing the net costs of introducing rotavirus vaccine by the incremental health benefits. ‘Incremental’ refers to the additional health benefits of introducing or implementing a new health intervention. We compare the implementation of a rotavirus vaccine program to the status quo, that is, relative to no vaccination. Table 3 presents a summary of the main study outputs, illustrating the impact of rotavirus vaccination over ten years beginning in 2017, accounting for a vaccine price of US$2.02 per dose.

**Table 3:** Summary of analysis outputs.

Key model outputs	Base case scenario	
	Without vaccine	With vaccine
Baseline rotavirus deaths (2017)	4880	3860
Baseline rotavirus admissions (2017)	20,069	15,874
Baseline rotavirus visits (2017)	269,898	213,479
Baseline rotavirus cases (2017)	497,999	393,897

*Model outputs with vaccination over 10 years, benefits and costs discounted*		
Cost per DALY averted [government; society]	US$82; US$80	
DALYs averted	651,283	
Cases averted	1.22 million	
Outpatient visits averted	661,746	
Inpatient visits averted	49,207	
Deaths averted	11,966	
Health costs averted (government)	US$1.35 million	
Health costs averted (societal)	US$2.8 million	
Vaccination program costs [with; w/o Gavi subsidy]	US$55 million; US$22 million	

In the base case analysis, the discounted cost per DALY averted is US$82 from the government perspective and US$80 from the societal perspective, making rotavirus vaccination a highly cost-effective intervention in Afghanistan relative to GDP per capita threshold of US$562 [31]. Afghanistan does not yet have its own cost-effectiveness threshold so we used one time per capita GDP as the threshold.

#### 3.1.1 Health impact

Table 4 reflects the projected discounted health benefits under the base case scenario with and without the vaccination program. Introduction of rotavirus vaccine in Afghanistan could result in a reduction of more than one million cases of rotavirus, prevention of approximately 650,000 DALYs, and would avert around 12,000 deaths by vaccinating over a 10-year period starting in 2017. Annual reduction of rotavirus cases and deaths would average 122,000 and 1200 respectively once the program is fully implemented.

**Table 4:** Discounted Health and economic benefits (10 cohorts vaccinated over the period of 2017–2026).

	No vaccine	With vaccine	Averted
*Health benefits*			
Cases	**5,045,332**	**3,824,319**	**1,221,013**
Non-severe	4,175,012	3,164,624	1,010,388
Severe	870,320	659,695	210,625
Visits	**2,734,395**	**2,072,648**	**661,746**
Non-severe	2,262,831	1,715,207	547,624
Severe	471,563	357,441	114,122
Hospitalization	**203,327**	**154,120**	**49,207**
Deaths	**49,444**	**37,478**	**11,966**
*Economic benefits*			
Government perspective	**US$5,639,844**	**US$4,286,135**	**US$1,353,709**
Visits	US$4,085,537	US$3,104,902	US$980,635
Hospitalizations	US$1,554,307	US$1,181,233	US$373,074
Societal perspective	**US$11,580,317**	**US$8,800,740**	**US$2,779,578**
Visits	US$8,764,901	US$6,661,097	US$2,103,805
Hospitalizations	US$2,815,416	US$2,139,643	US$675,773

Bold represents total numbers.

#### 3.1.2 Cost of care averted

Over the 10-year period, rotavirus vaccination would save the government US$1.35 million in treatment costs. Considering the societal perspective, accounting for household out-of-pocket costs, vaccination would avert around US$2.80 million (Table 4).

#### 3.1.3 Cost of vaccination program

In the base case scenario, the discounted cost of the vaccination program represents US$55 million for the 10-year period, or US$5.5 million per year on average. This estimate is considering that Afghanistan would have to pay the full price of the vaccine when, in reality, the country supports only a smaller share (the co-financing share) of the vaccine price. Accounting only for the co-financing amount of US$0.20 per dose and the delivery cost, the vaccination program cost is reduced to US$22 million for the 10-year period, or US$2.20 million per year on average. Additional details are provided in the alternative scenarios section.

#### 3.1.4 Alternative scenarios

**Fig. 1:**
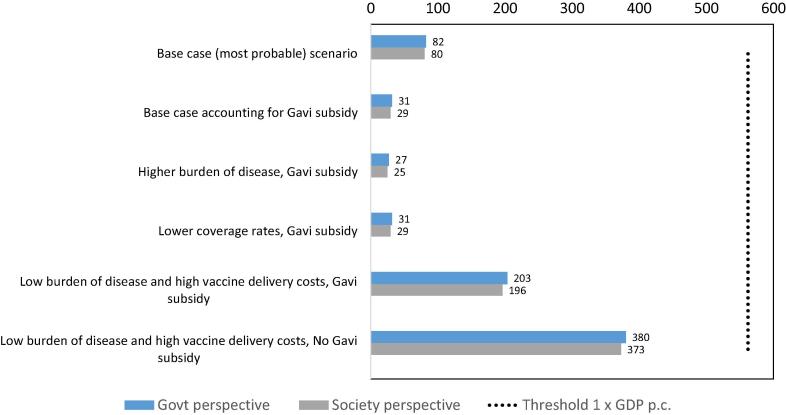
Discounted cost per DALY averted for all scenarios.

We examined five alternative scenarios, four of which use the vaccine price that Afghanistan is anticipated to pay. The range of ‘what if’ scenarios explored in the analysis cover both more and less favorable options and were informed by discussions with local experts (Table 5). When comparing results obtained for each alternative scenario to a threshold of one time GDP per capita for Afghanistan in 2016 [31], all scenarios prove to be highly cost-effective (ICER remains below US$562 GDP per capita) from the societal as well as from the government perspective. From the government perspective, the cost per DALY averted ranged from a minimum of US$27 for a scenario considering a higher disease burden (scenario 3) to a maximum of US$380 for our least favorable scenario, assuming a low burden of disease, higher vaccine delivery costs and no Gavi subsidy (scenario 6). From the societal perspective, the cost per DALY averted ranged from a minimum of US$25 to a maximum of US$373 for the same two scenarios (scenario 3 and 6 respectively). Fig. 1 shows the different ICERs per scenario for both the government and the societal perspectives.

**Table 5:** Description of alternative scenarios explored.

Scenario 1	Base case scenario	–
Scenario 2	Base case accounting for Gavi subsidy	Vaccine price lowered from US$2.02 per dose to US$0.20 to reflect co-financing amount paid by the country
Scenario 3	Higher burden of disease, Gavi subsidy	Incidence of rotavirus disease was increased by 15% for cases, visits, hospitalizations and deaths Vaccine price US$0.20 per dose
Scenario 4	Lower coverage rates, Gavi subsidy	Examining lower coverage rates in the first four years of the vaccination program to reflect 2015 DHS data Vaccine price US$0.20 per dose
Scenario 5	Low burden of disease and high vaccine delivery costs, Gavi subsidy	Examining a reduced burden of disease (cases, visits, and number of deaths based on IHME GBD data) Double incremental health system cost per dose (US$2.12 instead of initial US$1.06) Vaccine price US$0.20 per dose
Scenario 6	Low burden of disease and high vaccine delivery costs, No Gavi subsidy	Examining a reduced burden of disease (cases, visits, and number of deaths based on IHME GBD data) Double incremental health system cost per dose (US$2.12 instead of initial US$1.06) Vaccine price US$2.02 per dose

## 4 Discussion

This is the first formal analysis of the cost-effectiveness of rotavirus vaccine in Afghanistan. The analysis was conducted by members of the Afghanistan National Immunization Technical Advisory Group and Ministry of Public Health (MoPH). All input parameters were discussed and endorsed by a group of national and international experts. A strength of this study is the consensus achieved across several national stakeholders on the input parameters used and scenarios evaluated. The study has also helped to increase the capacity of the national team to conduct national economic evaluations for other vaccines in the future.

Based on recommendations made by the WHO Commission for Macroeconomics in Health [32], interventions were historically considered cost-effective, if the cost per DALY averted was less than three times a country’s GDP per capita, and highly cost-effective if the cost per DALY averted was less than one time GDP per capita. Recently WHO has updated these recommendations. Rather than using a uniform threshold based on per capita income, WHO now recommends that countries should establish their own specific thresholds while taking into consideration other factors such as affordability and feasibility [33]. While this new recommendation was discussed among the study group of experts, given the challenges for developing an appropriate threshold for Afghanistan, the study team decided to use one time the GDP per capita income, which is broadly consistent with the threshold used in Thailand [35] and far more conservative than the historical WHO recommendation (three times GDP per capita). In the base case scenario, excluding a Gavi subsidy, rotavirus vaccine would be highly cost-effective in Afghanistan, both from a societal and government perspective, with the ICER ranging from US$80 to US$82 per DALY averted. This finding is reinforced by the alternative scenarios, which include a scenario with multiple key inputs unfavourable to vaccination. There is limited information available about the potential cost-effectiveness of other interventions in Afghanistan, our estimates suggest that rotavirus vaccination would be at least as cost-effective, if not more so, than strategies to reduce maternal mortality [34].

Considering the support the country can receive from Gavi, the ICER ranges from US$29 to US$31 from the societal and government perspective, respectively. The average yearly cost of a rotavirus vaccination program would represent 2.8% of the total immunization cost expected in 2017 and 0.1% of the total health expenditures [23], [35].

One of the limitations of the study was the lack of high quality local data for some of the input parameters, for example, cost of hospital admission, rate of rotavirus mortality etc. However, we included internationally validated estimates in the model and tested for uncertainty around those estimates. In addition to discussions among the group of local experts, data inputs were validated by representatives of the MoPH, donors, international technical agencies, and local civil society organizations during a consultation workshop organized in Kabul, Afghanistan. Another important limitation is the exclusion of potential herd effects from the model. However, a recent effectiveness study in Bangladesh found limited evidence for herd effects [15] and inclusion of these benefits would only have made the case for vaccination stronger.

In summary, this analysis demonstrates that rotavirus vaccination is likely to be highly cost-effective and avert a substantial health and economic burden of disease in Afghanistan. The available evidence demonstrates that Afghanistan currently has one of the largest rotavirus disease burdens globally and rotavirus vaccination can have an enormous impact on this burden while minimally increasing the financial outlay. This study plays an important role in justifying Afghanistan’s decision to introduce rotavirus vaccination by demonstrating how impactful and cost-effective this intervention will be.

## Authors contribution

PA and FD helped conceptualize the study, led data collection and study analysis, interpreted results, drafted and revised the article.

CP, AC, SP contributed data, helped conceptualize the study, interpreted results, and contributed to article drafting.

DG participated in the study analysis and contributed to the drafting of the manuscript.

NS helped conceptualize the study and provided senior scientific support and oversight of the project and provided an essential link between the study and policy questions.

All authors revised and approved of the final version of this article.

## Source of funding

This work was supported by the Bill & Melinda Gates Foundation, Seattle, WA [grant number OPP1147721].

## Conflict of interest statement

The authors have no conflicts to declare.
